# Endogenous Reference Genes for Gene Expression Studies on Bicuspid Aortic Valve Associated Aortopathy in Humans

**DOI:** 10.1371/journal.pone.0164329

**Published:** 2016-10-11

**Authors:** Oliver J. Harrison, Narain Moorjani, Christopher Torrens, Sunil K. Ohri, Felino R. Cagampang

**Affiliations:** 1 Institute of Developmental Sciences, University of Southampton Faculty of Medicine, Southampton, United Kingdom; 2 Department of Cardiac Surgery, Papworth Hospital NHS Foundation Trust, Cambridge, United Kingdom; 3 Department of Cardiac Surgery, University Hospital Southampton, Southampton, United Kingdom; Harvard Medical School, UNITED STATES

## Abstract

Bicuspid aortic valve (BAV) disease is the most common congenital cardiac abnormality and predisposes patients to life-threatening aortic complications including aortic aneurysm. Quantitative real-time reverse transcription PCR (qRT-PCR) is one of the most commonly used methods to investigate underlying molecular mechanisms involved in aortopathy. The accuracy of the gene expression data is dependent on normalization by appropriate housekeeping (HK) genes, whose expression should remain constant regardless of aortic valve morphology, aortic diameter and other factors associated with aortopathy. Here, we identified an appropriate set of HK genes to be used as endogenous reference for quantifying gene expression in ascending aortic tissue using a spin column-based RNA extraction method. Ascending aortic biopsies were collected intra-operatively from patients undergoing aortic valve and/or ascending aortic surgery. These patients had BAV or tricuspid aortic valve (TAV), and the aortas were either dilated (≥4.5cm) or undilated. The cohort had an even distribution of gender, valve disease and hypertension. The expression stability of 12 reference genes were investigated (*ATP5B*, *ACTB*, *B2M*, *CYC1*, *EIF4A2*, *GAPDH*, *SDHA*, *RPL13A*, *TOP1*, *UBC*, *YWHAZ*, and *18S*) using geNorm software. The most stable HK genes were found to be *GAPDH*, *UBC and ACTB*. Both *GAPDH and UBC* demonstrated relative stability regardless of valve morphology, aortic diameter, gender and age. The expression of *B2M* and *SDHA* were found to be the least stable HK genes. We propose the use of *GAPDH*, *UBC and ACTB* as reference genes for gene expression studies of BAV aortopathy using ascending aortic tissue.

## Introduction

Bicuspid aortic valve (BAV) disease is the most common congenital heart condition affecting between 0.4%–2.25% of the worldwide population [1]. It results from abnormal formation of the aortic valve resulting in 2 valve cusps developing instead of the usual 3. The condition predisposes patients to premature valvulopathy (stenosis and regurgitation) and infective endocarditis [2]. Furthermore up to 80% of patients with BAV will develop enlargement of the ascending aorta increasing the risk of aortic rupture and dissection [3]. The collective term used to describe complications affecting the ascending aorta of BAV patients is ‘BAV aortopathy’. Mechanisms by which BAV aortopathy develops are not fully understood, however a combination of genetic predisposition and abnormal transvalvular haemodynamics are postulated [4]. Characteristic changes in the aortic wall distinguish BAV aortopathy. These include elastic laminar fragmentation, smooth muscle cell differentiation and apoptosis, matrix metalloproteinase dysregulation and accumulation of ground substance leading to degeneration of the tunica media [5, 6].

A common method for investigating underlying molecular mechanisms in BAV aortopathy is by quantifying gene expression using quantitative real-time reverse transcription PCR (qRT-PCR). This technique is especially suitable when small amounts of nucleic acids are available and provides simultaneous measurement of gene expression in several samples. However, the use of qRT-PCR requires compensation for differences between samples, which may be due to varying quality and quantity of the starting material. The expression levels of the genes of interest should therefore be normalized using endogenous control genes (reference genes), and some of the most frequently used reference genes are the constitutively expressed housekeeping (HK) genes. In order to accurately quantify any observed changes in gene expression, two or more validated HK genes should be used [7]. The essential prerequisites for all HK genes suitable for use as internal controls include being adequately expressed in the target tissue, and demonstrating minimal variability and high stability irrespective of physiological or pathological conditions. Previous studies have often utilised well known HK genes, such as glyceraldehyde-3-phosphatedehydrogenase (*GAPDH*) and 18s ribosomal RNA (*18S*) without validating their stability. Evidence from both aortic and non-aortic tissue studies suggests the stability of certain HK genes may vary significantly under differing experimental conditions, introducing the potential for misrepresentative data [8, 9]. Thus, it is recommended to validate potential HK genes within individual tissues and experimental systems before using them to normalize RT-qPCR data. To date, only two studies have investigated HK genes in ascending aortic tissue [9, 10]. Whilst a broad range of candidate HK genes were investigated, most of the HK genes analysed in the current study were not used and are thus potential novel HK genes for gene expression studies on the ascending aorta of BAV patients. In addition to BAV disease, several other factors may also influence gene expression and the risk of developing ascending aortic aneurysms (e.g. age, hypertension, hypercholesterolemia, use of cardiovascular medications) [11]. Furthermore, genetic, haemodynamic and cellular changes all have the potential to influence gene expression in context of BAV aortopathy. In the present study, we investigated 12 reference genes (*ATP5B*, *ACTB*, *B2M*, *CYC1*, *EIF4A2*, *GAPDH*, *SDHA*, *RPL13A*, *TOP1*, *UBC*, *YWHAZ*, and *18S*) in the ascending aortas of patients with different valve morphology, aortic diameters and other aortopathy risk factors, and used geNorm software to assess gene expression stability and determine how many reference genes were needed for accurate normalisation.

## Materials and Methods

### Patient selection

In accordance with the Declaration of Helsinki, all patients provided written, informed consent to participate in this study under a protocol approved by the local Ethics, Research, and Development Committee (NRES Committee South Central—Hampshire B, RHM CAR0392) and the Southampton University Hospitals NHS Trusts Research and Development department. Ascending aortic samples were collected intra-operatively from 22 patients undergoing aortic valve replacement and/or ascending aortic replacement. Ten patients had a tricuspid aortic valve (TAV) and 12 had a BAV. Both groups included a mix of dilated (≥4.5cm) and undilated (<4.5cm) ascending aortas, gender, hypertension and valve disease (Table 1). BAV patients tended to be younger than TAV patients but this was not statistically significant (*p* = 0.0738). Aortic valve morphology was confirmed intraoperatively, and ascending aortic diameter was calculated on perioperative trans-oesophageal echocardiography imaging.

**Table 1:** Patient demographics.

	BAV		TAV	
	Undilated	Dilated	Undilated	Dilated
**Sample Size (n)**	5	7	6	4
**Mean age ± SD**^a^ **(yrs)**	58.5±15.4	55.4±7.2	65.2±10.8	69.5±6.4
**Sex (M:F)**	3:2	5:2	2:4	2:2
**Hypertension (SBP**^b^**>140): Normotension**	3:2	1:6	2:4	2:2
**Valve disease**^c^	1xS, 2xR, 2xM	1xS, 1xR, 4xM, 1xN	6xS	3xR, 1xN
**Mean aortic diameter ± SDa (cm)**	4.0±0.3	5.5±0.4	3.5±0.5	6.2±1.3

^a^Standard deviation

^b^Systolic blood pressure

^c^S, stenosis; R, regurgitation; M, mixed; N, normal

### Tissue collection and processing

Tissue samples were collected intraoperatively. Where patients underwent isolated aortic valve replacement, a piece of tissue (approximately 5mm x 15mm in size) was excised from the aortotomy line prior to closure of the aorta. Samples were immediately snap frozen in liquid nitrogen and stored at -80°C until processing. Total RNA was extracted using RNeasy Fibrous Mini Kit (Qiagen, USA) from 30mg of pulverized aortic tissue. An on-column DNase step to remove genomic DNA was performed. Satisfactory RNA yield and quality was confirmed using a Nanodrop spectrophotometer (Thermoscientific, USA) and Agilent Bioanalyser (Agilent Technologies, USA). Reverse transcription was performed with GoScript Reverse Transcription kit (Promega, UK).

### Gene selection and data acquisition

The twelve candidate genes were included in the ‘Double-dye (Hydrolysis) probe geNorm 12 gene kit’ (PrimerDesign, UK). PrimerDesign selected these genes because they demonstrate stability across a number of different tissues [7]. The kit is particularly useful in the present study because it contained a mixture of genes that have been examined previously (e.g. *GAPDH*, *ACTB* and *UBC*) [9], and novel genes that have not yet been investigated in ascending aortic tissue. The candidate genes were adenosine triphosphate synthase (*ATP5B*), β-actin (*ACTB*), β-2-microglobulin (*B2M*), cytochrome c-1 (*CYC1*), eukaryotic translation initiation factor 4A isoform 2 (*EIF4A2*), *GAPDH*, succinate dehydrogenase complex subunit A (*SDHA*), ribosomal protein L13a (*RPL13A*) topoisomerase DNA I (*TOP1*), ubiquitin C (*UBC*), tyrosine 3-monooxygenase/tryptophan 5-monooxygenase activation protein, zeta polypeptide (*YWHAZ*) and *18S*. Messenger RNA expression levels were measured by qRT-PCR using primers and Perfect Probe (PrimerDesign, UK). Primer sequences are considered proprietary information and have not been disclosed by PrimerDesign. However, as per the MIQE guidelines primer position and sequence length are described in S1 Table [12]. Samples were analysed in duplicate using the Roche Lightcycler 480 PCR instrument (Roche Diagnostics Limited, UK). Identical optimised cycling conditions were used for all samples and Primer/Probe combinations combined with PrecisionPLUS Mastermix (PrimerDesign, UK). Plates were heated to 95°C for 2 min, then subject to 40 cycles of 60°C for 60 s and 95°C for 10 s. Cycle threshold (CT) values were recorded and entered directly into the geNorm software for analysis.

### Analysis of reference gene expression stability

Stability of the HK genes was calculated using a computer algorithm called geNorm [7] which compares expression variation between each of the candidate HK genes. As is described in a previously published paper from our group, qbasePLUS software version 3.4 (Biogazelle BE, Belgium) was used to process the transformed data [13]. From this, a stability measure (M) was generated by geometric averaging of multiple HK genes and mean pair-wise variation of a gene from all other HK genes for a given sample. It is based on the principle that an identical expression ratio between two ideal control genes will be observed in all samples independent of pathology and patient demographics. Genes with the lowest M values are deemed the most stable. As well as identifying the most stable and thus most suitable HK genes, the geNorm program introduces a pair-wise variation (V) which determines the optimum number of HK genes required for accurate normalization of gene expression. Pair-wise variation, V(n/n+1) determines the benefit gained from additional HK genes. A V score of 0.15 or below indicates that the additional gene has no significant contribution to the newly calculated normalization factor and is therefore not needed. In most cases, geNorm will recommend the use of two or three reference genes as a valid and accurate method of normalization strategy, compared with a single non-validated reference gene. The full PCR dataset used for analysis is available in S2 Table.

## Results

**Fig 1:**
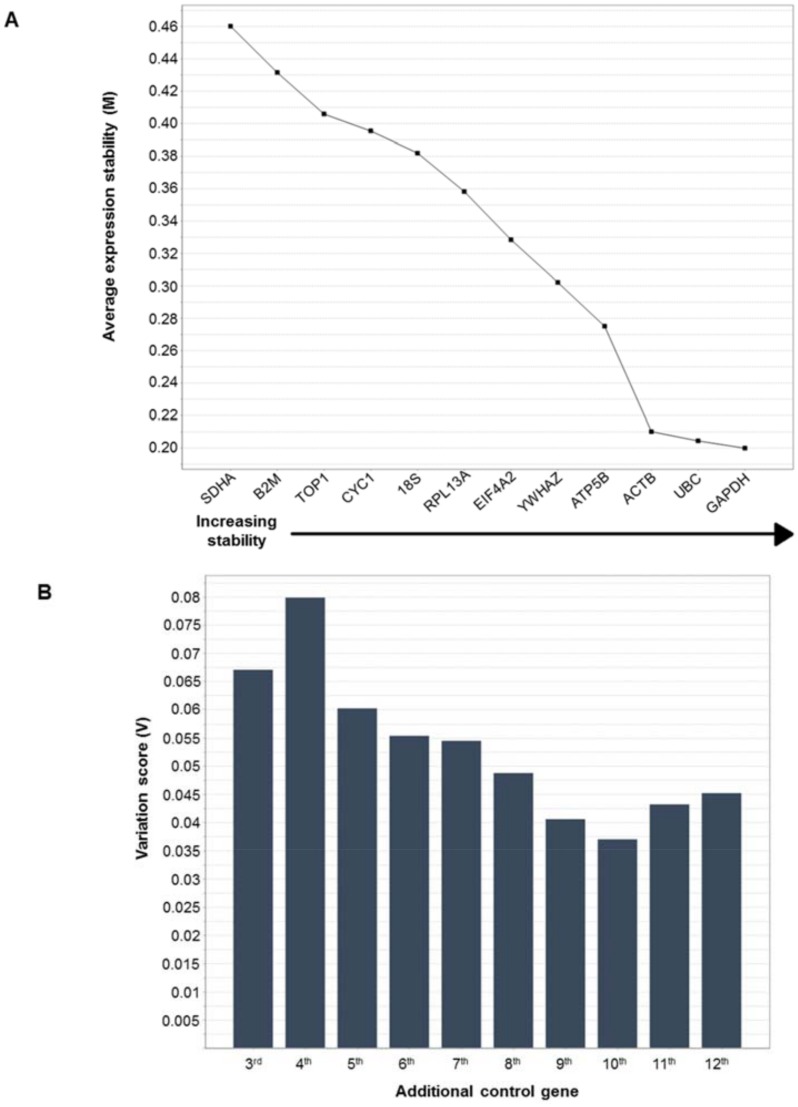
**(a) Expression stability of housekeeping (HK) genes.** The graph shows the average expression stability value (M) for each HK gene ranked according to increasing stability with the most stable genes (lowest M values) on the right (geNorm 3.4). **(b) The number of genes required for normalisation.** The graph shows the levels of variation in average HK gene stability with the addition of further housekeeping genes to the equation. The two most stable genes are included first with the inclusion of a 3rd, 4th, 5th gene etc. moving to the right (geNorm 3.4). The graph indicates that the two most stable genes created a normalization factor which is not significantly altered by the addition of more genes as they all have a V score below 0.15.

The 12 HK genes were ordered according to stability (Fig 1a) and the number of genes required for normalization determined (Fig 1b). The HK genes *GAPDH*, *UBC* and *ACTB* showed the highest stability when all samples were analyzed together, while *B2M* and *SDHA* were the least stable HK genes (Fig 1a). Nevertheless, all 12 HK genes had an M value below 1.5, which demonstrates relatively high reference gene stability in comparison to previous studies [9, 10]. The pair-wise variation (V) with the sequential addition of each reference gene indicated that the two most stable HK genes were sufficient as internal controls giving a normalization factor which was not significantly altered by the addition of the other HK genes since all had a V score below 0.15 (Fig 1b).

When samples were analyzed separately according to aortopathy or valve morphology (Table 2), *GAPDH*, *UBC* and *ACTB* were still shown to be the most stable HK genes in BAV and undilated patients. However, in TAV and dilated patients *GAPDH* and *UBC* were only moderately stable and *ACTB* became unstable. Conversely, *B2M* and *SDHA* remained the least stable HK genes irrespective of aortopathy or valve morphology, except that *SDHA* became moderately stable in dilated patients. When analyzing HK gene stability whilst comparing aortas from patients according to age and gender (Table 3), there was some disparity in stability of the HK genes. *GAPDH* and *UBC* remained stable or moderately stable regardless of gender or age, except *GAPDH* which became less stable in patients >60 years. *ACTB* was stable or moderately stable in younger males, but became unstable in older female patients. When the optimum number of HK genes was calculated separately according to aortopathy, valve morphology, patient’s age and gender, the two most stable HK genes were found to be sufficient as internal controls in all groups. All had a V score below 0.15 indicating that the additional gene would have no significant contribution to the normalization factor. As such athough *ACTB* is relatively stable, the two most stable genes *GAPDH* and *UBC* alone would be sufficient as internal controls.

**Table 2:** Expression levels and geNorm ranking and stability of HK genes in dilated and undilated ascending aortas from patients with a BAV and a TAV.

	TAV (n = 10)		BAV (n = 12)		Dilated (n = 11)		Undilated (n = 11)		Combined (n = 22)	
Gene	M- Value Stability^c^	Rank^a^	M- Value Stability^c^	Rank^a^	M- Value Stability^c^	Rank^a^	M- Value Stability^c^	Rank^a^	M- Value Stability^c^	Rank^b^
***18S***	0.209	5	0.415	8	0.212	3	0.358	9	0.382	8
***ATP5B***	0.148	1	0.308	6	0.176	1	0.234	5	0.275	4
***ACTB***	0.290	9	0.220	3	0.386	11	0.174	3	0.210	3
***B2M***	0.358	11	0.483	11	0.426	12	0.314	8	0.431	11
***CYC1***	0.236	6	0.443	9	0.330	8	0.383	10	0.395	9
***EIF4A2***	0.322	10	0.265	5	0.368	10	0.212	4	0.328	6
***GAPDH***	0.272	8	0.210	2	0.318	7	0.157	1	0.200	1
***RPL13A***	0.154	2	0.368	7	0.349	9	0.283	7	0.358	7
***SDHA***	0.396	12	0.506	12	0.252	4	0.447	12	0.460	12
***TOP1***	0.168	3	0.462	10	0.188	2	0.400	11	0.406	10
***UBC***	0.192	4	0.206	1	0.304	6	0.166	2	0.204	2
***YWHAZ***	0.250	7	0.287	6	0.270	5	0.256	6	0.302	5

^a^Stability ranking of 12 HK genes in dilated or undilated aortas in patients with a BAV or a TAV.

^b^Stability ranking of 12 HK genes irrespective of aortopathy and number of valve cusps.

^c^Each target gene underwent logarithmical transformation according to geNorm software to calculate a control gene-stability measure, M. This is defined as the pairwise variation of a particular gene with all other HK genes.

**Table 3:** Expression levels and geNorm ranking and stability of HK genes in age grouping and gender.

	Age				Gender			
	≤60 years (n = 10)		>60 years (n = 12)		Male (n = 12)		Female (n = 10)	
Gene	M- Value Stability^b^	Rank^a^	M- Value Stability^b^	Rank^a^	M- Value Stability^b^	Rank^a^	M- Value Stability^b^	Rank^a^
***18S***	0.428	9	0.216	4	0.426	9	0.240	1
***ATP5B***	0.294	6	0.251	6	0.215	4	0.312	9
***ACTB***	0.271	5	0.290	10	0.160	1	0.371	12
***B2M***	0.317	7	0.356	12	0.483	11	0.324	10
***CYC1***	0.462	10	0.232	5	0.403	8	0.269	5
***EIF4A2***	0.221	1	0.158	1	0.313	6	0.259	4
***GAPDH***	0.250	4	0.279	9	0.173	2	0.290	7
***RPL13A***	0.375	8	0.263	7	0.367	7	0.245	2
***SDHA***	0.518	12	0.324	11	0.520	12	0.346	11
***TOP1***	0.486	11	0.171	3	0.444	10	0.300	8
***UBC***	0.229	3	0.162	2	0.179	3	0.281	6
***YWHAZ***	0.223	2	0.269	8	0.251	5	0.252	3

^a^Stability ranking of 12 HK genes in BAV and TAV patients with dilated and undilated aortas according to age and gender

^b^Each target gene underwent logarithmical transformation according to geNorm software to calculate a control gene-stability measure, M. This is defined as the pairwise variation of a particular gene with all other HK genes.

## Discussion

The present study has demonstrated that the HK genes *GAPDH* and *UBC* are the most appropriate reference genes for accurate normalization of gene expression when investigating BAV aortopathy. Additionally the expression stability of these two HK genes alone is sufficient for their geometric means to be used for normalization of gene expression. In previous studies, the stability of *GAPDH* as a reference gene has been questioned, particularly in the setting of tissues and cells exposed to hypoxia [14]. Furthermore, in human colon, lung, brain and heart tissue, *GAPDH* expression appears variable, again in some cases apparently through mechanisms linked to hypoxia [15–18]. This may explain why in the relatively well-perfused ascending aorta *GAPDH* expression remains relatively stable. Interestingly however, whilst *GAPDH* remained relatively stable across all experimental groups it became less stable in patients >60 years, TAV patients and dilated aortas. We hypothesise that in older TAV patients with dilated aortas, atherosclerosis and tissue hypoxia may be more prevalent which may explain why *GAPDH* becomes less stable in these groups. In support of this observation a previous study demonstrating low stability of *GAPDH* in ascending aortic tissue sampled a higher proportion of TAV patients compared to the present study (58% versus 45% respectively) [9].

The only other study to date that investigated stability of *GAPDH* in ascending aortic tissue suggested that this HK gene was a relatively poor reference gene [9]. An important difference with the previous study is that RNA extraction was performed using a Trizol-based method rather than the spin column method used in the present study. Differing methods of RNA extraction have previously been shown to influence the stability and suitability of HK genes [19]. This may explain why *GAPDH* and *UBC* were found to be most stable in the present study, and these genes may represent a more appropriate HK choice when using a spin column method to extract RNA from ascending aortic tissue. Furthermore, the mean age of patients used in the present study was higher and the ratio of males to females was lower compared to previous studies [9, 10]. These factors may have contributed to the differing stability of the HK genes identified.

Consistent with previous studies [9], we found the HK gene *UBC* to be a stable reference gene in ascending aortic tissue. However, we also found *ACTB* to be relatively stable which is on contrast to this previous study. The disparity between the present finding and those from previous studies [9, 15–18] in terms of stability of *GAPDH* highlights the importance of confirming the stability of HK genes in any gene expression studies. There are inherent variations in gene expression due to factors such as the amount or quality of starting material, enzymatic efficiencies, and differences between tissues or cells. Thus, relying solely on previously validated HK genes even in the same tissue and disease type can introduce errors to data interpretation. Similarly to previous studies we were unable to obtain tissue from healthy ‘non-cardiac’ patients to represent a true control group. We used TAV patients undergoing cardiac surgery as a control for comparison to BAV. However, HK gene stability may have been different in aortic tissue taken from younger patients with no cardiac disease. We also had a relatively small number of patients in comparison to previous studies. However, despite these limitations all the 12 HK genes examined had low M values, which demonstrate relatively high reference gene stability in comparison to what others have found in previous studies.

In summary, we found *GAPDH*, *UBC* and *ACTB* to be the most stable HK genes in the ascending aortic tissues from patients with both TAV and BAV, and dilated and non-dilated aortas. Housekeeping gene stability appears to be affected by RNA extraction method and we recommend using *GAPDH*, *UBC* and *ACTB* with a spin column extraction method for normalization of gene expression when investigating mechanisms involved in BAV aortopathy.

## Supporting Information

S1 Table Raw PCR data. Data are listed numerically according to study ID together with the PCR plate well, the crossing threshold (Ct) value and corresponding candidate housekeeping gene.
(XLSX)

S2 Table Primer information supplied by PrimerDesign. (XLSX)

## References

[1] Hoffman JIE, Kaplan S. The incidence of congenital heart disease. Journal of the American College of Cardiology. 2002;39(12):1890–900. 10.1016/S0735-1097(02)01886-7. 12084585

[2] Tzemos N, Therrien J, Yip J, Thanassoulis G, Tremblay S, Jamorski MT, et al Outcomes in adults with bicuspid aortic valves. JAMA. 2008;300(11):1317–25. Epub 2008/09/19. 10.1001/jama.300.11.1317 . 18799444

[3] Della Corte A, Bancone C, Quarto C, Dialetto G, Covino FE, Scardone M, et al Predictors of ascending aortic dilatation with bicuspid aortic valve: a wide spectrum of disease expression. European journal of cardio-thoracic surgery: official journal of the European Association for Cardio-thoracic Surgery. 2007;31(3):397–404; discussion -5. 10.1016/j.ejcts.2006.12.006 . 17236783

[4] Abdulkareem N, Smelt J, Jahangiri M. Bicuspid aortic valve aortopathy: genetics, pathophysiology and medical therapy. Interactive cardiovascular and thoracic surgery. 2013;17(3):554–9. 10.1093/icvts/ivt196 23728086 PMC3745132

[5] Pisano C, Maresi E, Balistreri CR, Candore G, Merlo D, Fattouch K, et al Histological and genetic studies in patients with bicuspid aortic valve and ascending aorta complications. Interactive cardiovascular and thoracic surgery. 2012;14(3):300–6. 10.1093/icvts/ivr114 22194275 PMC3290383

[6] Majumdar R, Miller DV, Ballman KV, Unnikrishnan G, McKellar SH, Sarkar G, et al Elevated expressions of osteopontin and tenascin C in ascending aortic aneurysms are associated with trileaflet aortic valves as compared with bicuspid aortic valves. Cardiovascular pathology: the official journal of the Society for Cardiovascular Pathology. 2007;16(3):144–50. 10.1016/j.carpath.2006.12.001 . 17502243

[7] Vandesompele J, De Preter K, Pattyn F, Poppe B, Van Roy N, De Paepe A, et al Accurate normalization of real-time quantitative RT-PCR data by geometric averaging of multiple internal control genes. Genome biology. 2002;3(7):Research0034 Epub 2002/08/20. ; PubMed Central PMCID: PMCPmc126239. 12184808 10.1186/gb-2002-3-7-research0034 PMC126239

[8] Schmittgen TD, Zakrajsek BA. Effect of experimental treatment on housekeeping gene expression: validation by real-time, quantitative RT-PCR. Journal of biochemical and biophysical methods. 2000;46(1–2):69–81. Epub 2000/11/22. 10.1016/S0165-022X(00)00129-9 . 11086195

[9] Henn D, Bandner-Risch D, Perttunen H, Schmied W, Porras C, Ceballos F, et al Identification of Reference Genes for Quantitative RT-PCR in Ascending Aortic Aneurysms. PloS one. 2013;8(1):e54132 10.1371/journal.pone.0054132 23326585 PMC3543309

[10] Rueda-Martinez C, Lamas O, Mataro MJ, Robledo-Carmona J, Sanchez-Espin G, Jimenez-Navarro M, et al Selection of reference genes for quantitative real time PCR (qPCR) assays in tissue from human ascending aorta. PloS one. 2014;9(5):e97449 Epub 2014/05/21. 10.1371/journal.pone.0097449 ; PubMed Central PMCID: PMCPmc4026239. 24841551 PMC4026239

[11] Isselbacher EM. Thoracic and abdominal aortic aneurysms. Circulation. 2005;111(6):816–28. Epub 2005/02/16. 10.1161/01.cir.0000154569.08857.7a . 15710776

[12] Bustin SA, Benes V, Garson JA, Hellemans J, Huggett J, Kubista M, et al The MIQE guidelines: minimum information for publication of quantitative real-time PCR experiments. Clin Chem. 2009;55(4):611–22. Epub 2009/02/28. 10.1373/clinchem.2008.112797 . 19246619

[13] Cleal JK, Shepherd JN, Shearer JL, Bruce KD, Cagampang FR. Sensitivity of housekeeping genes in the suprachiasmatic nucleus of the mouse brain to diet and the daily light—dark cycle. Brain Research. 2014;1575:72–7. 10.1016/j.brainres.2014.05.031. 24881883

[14] Caradec J, Sirab N, Keumeugni C, Moutereau S, Chimingqi M, Matar C, et al 'Desperate house genes': the dramatic example of hypoxia. British journal of cancer. 2010;102(6):1037–43. Epub 2010/02/25. 10.1038/sj.bjc.6605573 ; PubMed Central PMCID: PMCPmc2844028. 20179706 PMC2844028

[15] Glare EM, Divjak M, Bailey MJ, Walters EH. beta-Actin and GAPDH housekeeping gene expression in asthmatic airways is variable and not suitable for normalising mRNA levels. Thorax. 2002;57(9):765–70. Epub 2002/08/30. 10.1136/thorax.57.9.765 ; PubMed Central PMCID: PMCPmc1746418. 12200519 PMC1746418

[16] Tricarico C, Pinzani P, Bianchi S, Paglierani M, Distante V, Pazzagli M, et al Quantitative real-time reverse transcription polymerase chain reaction: normalization to rRNA or single housekeeping genes is inappropriate for human tissue biopsies. Analytical biochemistry. 2002;309(2):293–300. Epub 2002/11/05. 10.1016/S0003-2697(02)00311-1 . 12413463

[17] Pilbrow AP, Ellmers LJ, Black MA, Moravec CS, Sweet WE, Troughton RW, et al Genomic selection of reference genes for real-time PCR in human myocardium. BMC medical genomics. 2008;1:64 Epub 2008/12/31. 10.1186/1755-8794-1-64 ; PubMed Central PMCID: PMCPmc2632664. 19114010 PMC2632664

[18] Pfister C, Tatabiga MS, Roser F. Selection of suitable reference genes for quantitative real-time polymerase chain reaction in human meningiomas and arachnoidea. BMC research notes. 2011;4:275 Epub 2011/08/03. 10.1186/1756-0500-4-275 ; PubMed Central PMCID: PMCPmc3166272. 21806841 PMC3166272

[19] Cleal JK, Day P, Hanson MA, Lewis RM. Measurement of housekeeping genes in human placenta. Placenta. 2009;30(11):1002–3. Epub 2009/10/13. 10.1016/j.placenta.2009.09.002 . 19819546

